# Burden of tuberculosis disease among adolescents in a rural cohort in Eastern Uganda

**DOI:** 10.1186/1471-2334-13-349

**Published:** 2013-07-26

**Authors:** James Waako, Suzanne Verver, Anne Wajja, Willy Ssengooba, Moses L Joloba, Robert Colebunders, Philippa Musoke, Harriet Mayanja-Kizza

**Affiliations:** 1 Makerere University Iganga/Mayuge Demographic Surveillance Site, P.O BOX. 22418, Kampala, Uganda; 2 KNCV Tuberculosis Foundation, The Hague, and CINIMA, Academic Medical Centre Amsterdam, Amsterdam, The Netherlands; 3 Makerere University Infectious Diseases Institute, Kampala, Uganda; 4 Department of Microbiology, Makerere University College of Health Sciences, Kampala, Uganda; 5 Institute of Tropical Medicine, Antwerp, Belgium; 6 University of Antwerp, Antwerp, Belgium; 7 Department of Paediatrics and Child Health, Makerere University College of Health Sciences, Kampala, Uganda; 8 Department of Medicine, Makerere University College of Health Sciences, Kampala, Uganda

## Abstract

**Background:**

The world health organization (WHO) declared tuberculosis (TB) a global emergency, mainly affecting people in sub-Saharan Africa. However there is little data about the burden of TB among adolescents. We estimated the prevalence and incidence of TB and assessed factors associated with TB among adolescents aged 12–18 years in a rural population in Uganda in order to prepare the site for phase III clinical trials with novel TB vaccines among adolescents.

**Methods:**

In a prospective cohort study, we recruited 5000 adolescents and followed them actively, every 6 months, for 1–2 years. Participants suspected of having TB were those who had any of; TB signs and symptoms, history of TB contact or a positive tuberculin skin test (TST) of ≥10 mm. Laboratory investigations included sputum smear microscopy and culture.

**Results:**

Of the 5000 participants, eight culture confirmed cases of TB were found at baseline: a prevalence of 160/100,000 (95% confidence interval (CI), 69–315). There were 13 incident TB cases detected in an average of 1.1 person years: an incidence of 235/100,000 person years (95% CI, 125–402). None of the confirmed TB cases were HIV infected. Predictors for prevalent TB disease were: a history of TB contact and a cough ≥ 2 weeks at baseline and being out of school, while the only predictor for incident TB was a positive TST during follow-up.

**Conclusion:**

The TB incidence among adolescents in this rural part of Uganda seemed too low for a phase III TB vaccine trial. However, the study site demonstrated capability to handle a large number of participants with minimal loss to follow-up and its suitability for future clinical trials. Improved contact tracing in TB program activities is likely to increase TB case detection among adolescents. Future studies should explore possible pockets of higher TB incidence in urban areas and among out of school youth.

## Background

Tuberculosis (TB) is a global emergency causing high morbidity and mortality especially in sub-Saharan Africa [1]. The immune response against Mycobacterium tuberculosis (MTB) is less effective among adolescents [2]. Adolescents have peculiar clinical TB manifestations and may present without symptoms but are more likely to have cavitating disease compared to adults or children [3]. Vaccination against TB using Bacillus Calmette-Guerin (BCG) in neonates has led to a reduction in incidence of severe childhood TB and deaths including miliary TB and TB meningitis [4]. Despite BCG immunization, adult forms of TB continue to emerge implying the current vaccine has limited efficacy against adult TB and therefore a need for new TB vaccines with high protective efficacy [5-7]. The risk of developing TB following infection starts to rise from 12–19 years making it a suitable age group for new TB vaccines [8].

Diagnosed TB among adolescents in most countries is aggregated and reported together with adult TB cases, disaggregated as 0–14 and 15–25 years, with limited information specific to adolescents. Very few studies have investigated TB prevalence and incidence of TB among adolescents [9]. Uganda is ranked 16th among the 22 TB high burden countries globally, with estimated incidence rates of about 209 per 100,000 population per year for all forms of TB in 2010 [10]. The estimated prevalence was 193/100,000 population for all forms of TB in 2010. However, the country notified an incidence of 128/100,000 population, only 66% of the estimated incidence [1].

The objectives of the study were to estimate the prevalence and incidence of TB and assess factors associated with TB among adolescents aged 12–18 years in a rural population in Uganda in order to prepare the site for phase III clinical trials with novel TB vaccines among adolescents.

## Methods

The study was conducted in the Makerere University School of Public Health Demographic Surveillance Site (DSS) in Iganga and Mayuge districts, located 120 km east of Kampala between September 2009 and September 2011. The study area is 80% rural and 20% peri-urban with nine health facilities offering free TB smear microscopy and treatment. The study area had a population of approximately 12,000 adolescents aged 12–18 years.

Participants aged 12–18 years who were resident in the study area for the previous three months were approached both in school and at home. Those who gave written assent after the parents/legal guardian had provided written informed consent were enrolled into the study. Participants who were planning to move away from the study area within one year were excluded.

Adolescents were stratified into 12–14 years, 15–16 years and 17–18 year age groups and conveniently sampled from schools and in the community with the sample size in each age stratum proportional to the size of the stratum population in the DSS. They were recruited consecutively until the sample size of 5000 was attained. Recruitment was mainly from rural areas and all had an equal chance of inclusion in the study. Given the World Health Organization (WHO) estimated incidence for 2007 of 330/100,000 population at time of study start (September 2009) [10] and 10-20% loss to follow-up; we expected to find among 5000 adolescents a cumulative TB incidence of 28 (0.56%) cases in two years, with a 95% CI of {26 (0.37%) - 51 (0.73%)}. These estimates were amended by WHO downwards in the later reports.

At enrolment, socio-demographic variables, relevant medical history including symptoms and signs suggestive of TB were collected. The participant’s weight and temperature were taken and a physical examination done. All participants had a tuberculin skin test (TST) 2 Tuberculin Units (Danish type from Staten’s Serum Institute) placed and read after 48–72 hours. Those with a negative result had a repeat TST done at one and two years of follow up. Participants were followed up every 6 months for 1–2 years and targeted history and physical examination were done. The study closed early and therefore those recruited later had a shorter duration of follow-up. Active TB suspicion was defined as participants found with any one of the following; TST induration of ≥10 mm, or at least one TB related symptom: cough ≥ 2 weeks, weight loss for ≥2 weeks, fever for ≥2 weeks, haemoptysis) or a history of TB contact since the last study follow up visit. Two sputum samples, a spot sample on day one and an early morning sample on day 2 were collected from all TB suspects and transported in cold boxes (temperature ≤8°C) to the reference laboratory within 24 hours. All collected data were entered onto a pre-coded case report form (CRF).

### Laboratory analysis

Sputum samples were processed in the Makerere University mycobacteriology Biosafety Level III laboratory in Kampala. All sputum samples were subjected to fluorescence microscopy and culture after concentration using 1% sodium hydroxide (NAOH)/N-acetyl L-cysteine (NALC) and sodium citrate method [11]. Cultures were performed by both solid (Lowenstein-Jensen, (Becton and Dickson, Franklin Lakes, NJ USA)) and liquid Mycobacterium Growth Indicator Tube (MGIT) culture techniques and incubated for six to eight weeks. Species identification was done using Capilia TB Neo™ (TAUN, Numazu, Japan) assay for all Ziehl-Neelsen (ZN) positive cultures to exclude mycobacteria other than tuberculosis (MOTT) and all isolates of mycobacterium tuberculosis complex (MTBC) were subjected to Hain Genotype MTBC assay for speciation. Participants with at least two positive smears or at least one MTBC culture were defined as confirmed TB cases.

### Ethical approval

Ethical approval was obtained from the Makerere University School of Public Health Higher Degrees Research and Ethics Committee, the Uganda National Council for Science and Technology and the Institutional Review Board of Institute of Tropical Medicine (ITM) Antwerp of Belgium.

### Data analysis

Analysis was done using STATA statistical software (Release 11.0, Stata Corporation, College Station, Texas, United States of America). Primary analysis was done by calculating frequencies for key demographic variables and overall prevalence and incidence rates; with secondary analyses done to estimate stratum specific prevalence and incidence rates by age group, sex, education, schooling status, TST outcome, TB contact and cough. Person years were calculated by subtracting the day of entry into the study from the last day of contact with the participant divided by 365 days. For those diagnosed with TB, date of diagnosis was taken as date of last contact. For those who died, date of death was taken as last date of contact. 95% confidence intervals (CI) were calculated using Poisson distribution. Odds ratios (OR) were calculated as a measure of TB risk and hazard ratios were calculated for incident TB cases. In multivariate analysis we included variables that were significant in univariate analysis at the P < 0.01 level.

## Results

**Figure 1:**
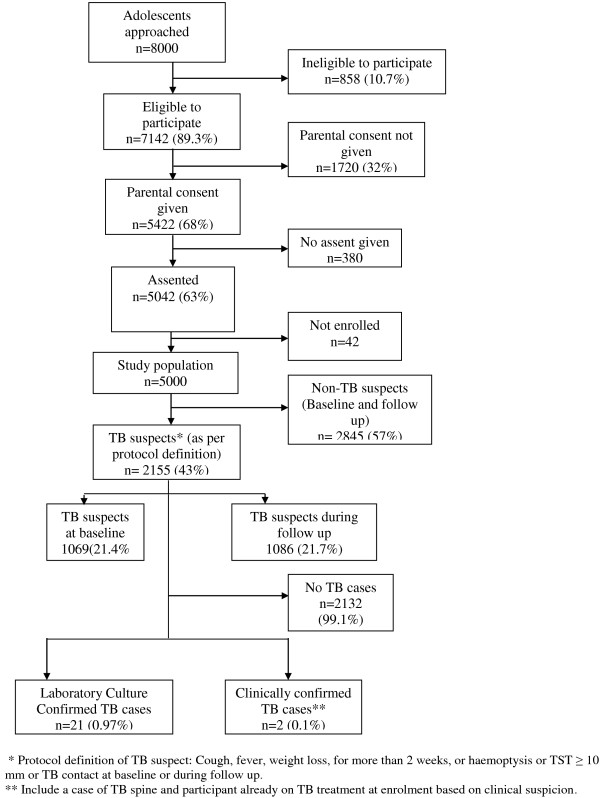
Study profile and TB cases among adolescents in a rural cohort in eastern Uganda.

Among the DSS adolescents; 8000 were approached and 5422 (68%) parents agreed to have their children participate in the study and 5042 (63%) adolescents provided assent. Among the screened participants, 858 (10.7%) were ineligible to participate mainly because they were planning to move away from the study area within one year. A total of 5000 adolescents were consecutively enrolled over a 12 month period. Of these, 1069(21.4%) were TB suspects at baseline and 1086 (21.7%) during follow-up. The most common reasons were, a positive TST 1307 (54.7%), symptoms suggestive of TB 735 (30.8%) and a history of TB contact 441 (18.5%), Figure 1.

### Description of study participants

Study participants were more often male (52%); unmarried (99%), aged 12–14 years (47%), Basoga (82%) the predominant tribe in the area and majority were in school (94%), Table 1. A total of 12,124 follow up visits were planned of which 10,769 (89%) were conducted. The follow up period was at least 12 months and maximum 24 months with an overall loss to follow up of 10.5% and total person years of follow up was 5527 with mean follow up time of 1.1 person years (SD 0.3). Four participants died of: juvenile diabetes, tetanus, sickle cell anemia and a possible cardiac problem and none was due to TB. A total of 143/5000 (3%) participants were never seen again after enrollment.

**Table 1:** Baseline characteristics of adolescents enrolled in the Iganga/Mayuge TB Cohort Study, 2009

**Characteristic**	**Study population**	**Percentage**
***Total***	***5000***	***100***
**Age (Years)**		
12-14	2339	47
15-16	1482	29
17-18	1179	24
**Sex**		
Male	2589	52
**Marital status**		
Married	33	0.7
**Level of education**		
>7 years	696	13.9
≤ 7 years	4250	85
Never attended school	14	0.3
Unknown	40	0.8
**Enrolment status**		
In school	4678	94
Out of school	322	6
**Tribe**		
Basoga	4089	81.8
Others	911	18.2

### TB prevalence and incidence

A total of nine TB cases out of 5000 adolescents were detected at baseline of which eight cases were confirmed microbiologically, Table 2. Of these eight, seven were smear (at least 1) and culture positive, and one was smear negative but culture positive. One of the TB cases was identified while on TB treatment (smear and culture positive). This resulted in a crude prevalence of culture confirmed TB of 160/100,000 (95% CI, 69–315). Of these eight cases, four had only TB symptoms, three had only a history of TB contact and one had both symptoms and a TB contact. One participant was clinically diagnosed with TB in the health facility. This participant was HIV infected, had TB symptoms and history of TB contact, but sputum samples were negative on smear microscopy and culture, Table 2. There were no prevalent TB cases among adolescents with a negative TST.

**Table 2:** Clinical and laboratory characteristics of prevalent TB cases (n = 9)

**Case number**	**TB symptoms**	**TB contact**	**TST** ≥**10 mm**	**CXR abnormalities present**	**Smear positive**	**Culture positive**	**Case determination criteria**
1.	No	Yes	Yes	Yes	Yes	Yes	Smear and culture
2.	Yes	No	Yes	No	Yes	Yes	Smear and culture
3.	Yes	No	Yes	Yes	Yes	Yes	Smear and culture
4.	Yes	No	Yes	Yes	Yes	Yes	Smear and culture
5.	No	Yes	Yes	No	Yes	Yes	Smear and culture
6.	No	Yes	Yes	Yes	Yes	Yes	Smear and culture
7.	Yes	No	Yes	No	Yes	Yes	Culture only
8.	Yes	Yes	Yes	Yes	Yes	Yes	Smear and culture
9. ***	Yes	Yes	Yes	Yes	No	No	Clinical diagnosis
Total	6	5	9	6	8	8	

*** HIV positive participant.

Abbreviation: Key: *TB* Tuberculosis, *TST* Tuberculin Skin Test, *CXR* Chest X-Ray.

During follow up, 14 cases of TB were detected of which 13 were culture confirmed (all smear negative) and one was clinically diagnosed with TB. The average duration of follow up was 1.1 years. The incidence of bacteriologically confirmed TB was 235/100,000 person years (95% CI, 125–402). All TB cases were HIV negative. Nine (64%) reported no symptoms and none had CXR abnormalities (CXR was done for only nine cases after start of treatment for clinical care purposes), Table 3.

**Table 3:** **Clinical and laboratory characteristics of the adolescent incident TB cases** (**n** = **14)**

**Case number**	**Visit detected (months)**	**TB symptoms**	**TB contact**	**TST** ≥**10 mm**	**CXR abnormalities**	**Smear positive**	**Culture positive**	**Case determination criteria**^**1**^
1	12	Yes	No	Yes	Not done	No	Yes	Culture
2	6	Yes	No	Yes	Yes**	No	No	Clinical diagnosis▲
3	12	No	No	Yes	No	No	Yes	Culture
4	12	No	No	Yes	No	No	Yes	Culture
5	12	No	Yes	Yes	No	No	Yes	Culture
6	12	No	Yes	Yes	No	No	Yes	Culture
7	12	No	No	Yes	No	No	Yes	Culture
8	12	No	No	Yes	Not done	No	Yes	Culture
9	12	No	No	Yes	Not done	No	Yes	Culture
10	12	Yes	No	Yes	Not done	No	Yes	Culture
11	24	No	No	Yes	No	No	Yes	Culture
12	24	No	No	Yes	No	No	Yes	Culture
13	12	Yes	No	Yes	No	No	Yes	Culture
14	12	Yes	No	Yes	Not done	No	Yes	Culture
Total		5	2	14	None	None	13	14

▲POTTS disease.

^1^ Culture - all smears were negative.

** Necrosis and collapse of thoracic vertebrae 10–12.

Abbreviation: Key: *TB* Tuberculosis, *TST* Tuberculin Skin Test, *CXR* Chest X-Ray.

### Predictors for prevalent TB

At baseline there was no significant difference in adolescents with bacteriologically confirmed TB by sex, age groups, and levels of education. Participants who were out of school were seven times more likely to have TB than their in-school counter parts (AOR 6.84; 01.28-36.03). Participants who had contact with a TB patient were sixteen times more likely to have TB compared to those with no TB contact (AOR 15.67; 3.57 -68.77). Those who had had cough for ≥14 days were five times more likely to have TB than those who had had cough for ≤14 days or no cough at all (AOR 5.15; 1.20-22.10), Tables 4 and 5.

**Table 4:** Univariate predictors of prevalent TB

**Background characteristic**	**TB cases n = 8**	**Population N = 5000**	**Prevalence/100,000**	**P-value (Chi-square test)**
**Gender**				
Male	3	2589	116	0.419
Female	5	2411	207	
**Age (yrs)**				
12-14	4	2339	171	
15-16	3	1482	202	0.74
17-18	1	1179	85	
**Schooling status**				
Out of school	2	322	621	0.032
In school	6	4678	128	
**Level of education**				
> 7 years	1	696	144	
≤ 7 years	7	4264	164	0.961
Unknown	0	40	0	
**Baseline TST**				
Positive (≥10 mm)	8	802	998	
Negative (<10 mm)	0	4179	0	<0.001
Not done / Not Read	0	19	0	
**TB contact**				
Yes	4	237	1409	0.115
No	4	4763	76	
**Cough duration (ever)**				
Cough <14 days	3	2548	118	
No cough	0	1827	0	<0.001
Cough ≥14 days	5	625	800	

Abbreviation: Key: *TST* Tuberculin Skin test.

**Table 5:** Associations between patient characteristics and prevalence of TB

**Background characteristic**	**Unadjusted OR**	**95% CI**	**Adjusted OR**	**95% CI**
**Gender**				
Male	Reference		Reference	
Female	1.79	0.43 - 7.50	1.74	0.42 - 7.30
**Age (yrs)**				
12-14	Reference		Reference	
15-16	1.18	0.27 - 5.30	0.95	0.12 - 7.47
17-18	0.5	0.06 - 4.34	0.27	0.02 - 3.72
**Schooling status**				
Out of school	4.87	0.98 - 24.21	6.8	1.28 - 36.03
In school	Reference		Reference	
**Level of education**				
> 7 years	Reference		Reference	
≤ 7 years	1.14	0.14 - 9.30	0.63	0.03 - 13.23
Unknown	--		--	
**TB contact (baseline)**				
Yes	20.42	5.08 - 82.18	15.67	3.57 - 68.77
No	Reference		Reference	
**Cough duration (ever)**				
Cough < 14 days	Reference		Reference	
No cough	--		--	
Cough ≥ 14 days	6.84	1.63 - 28.70	5.15	1.20 - 22.10

Abbreviation: Key: 95% *CI* 95% Confidence Interval.

### Predictors for incident TB

On multivariate analysis, bacteriologically confirmed TB incidence during follow up was significantly higher among participants with a positive TST ≥10 mm than their TST negative counter parts ( adjusted hazard ratio 58.08; 95% CI 7.2-435). The incidence of TB seemed to increase with age though this trend was not significant, p-value = 0.093. Incidence was similar among those with ≥ seven years of schooling compared to those with lower education level (Adjusted hazard ratio 0.9; 0.20, 4.06). Sex specific incidence rates showed males being three times more likely to develop TB than females though this difference was also not statistically significant (Adjusted hazard ratio 0.32: 0.09-1.17). TB incidence was higher among those who had TB contact and those who had coughed for ≥14 days, but this was not significant. Those with incident TB had no prior diagnosis of TB and were all school-going participants, Tables 6 and 7.

**Table 6:** Univariate predictors of incident TB

**Determinant**	**Persons**	**Pyrs**	**Cases**	**Incidence (per 100,000 pyrs)**	**P-value (chi-square test)**
**Total**	**5000**	**5527**	**13**	**235**	**-**
**Follow up TST outcome** *					
Negative < 10 mm	2912	3323	1	30	
Positive ≥ 10 mm	582	679	11	1620	<0.001
Not done/not read**	1506	1525	1	66	
**Baseline TST outcome**					
Negative < 10 mm	4179	4574	12	262	<0.001
Positive ≥ 10 mm	802	890	1	112	
**Prior diagnosis TB**					
No	4990	5517	13	236	-
Yes	10	11	0	0	
**Schooling status**					
In school	4678	5193	13	250	-
Out of school	322	335	0	0	
**Age group (years)**					
12-14	2339	2778	4	144	
15-16	1482	1583	4	253	0.093
17-18	1179	1167	5	429	
**Sex**					
Male	2589	2898	10	345	
Female	2411	2630	3	114	0.028
**Education level**					
> 7 years	696	733	2	273	
None	40	37	0	0	0.752
≤ 7 years	4264	4757	11	231	
**TB Contact during follow-up**					
No	4732	5196	11	212	0.019
Yes	268	331	2	604	
**Cough during follow-up**					
<14 days	2250	2602	5	192	
None	2102	2307	6	260	0.917
≥14 days	500	581	2	344	

Abbreviation: Key: *Pyrs* Person years, *TST* Tuberculin Skin Test.

Note:

* One individual with TB during follow up had a positive TST of ≥10 mm at baseline;

**Those with a positive TST at baseline were not given TST again at 1 year and 2 years of follow up.

**Table 7:** Associations between patient characteristics and incidence of TB

**Determinant**	**Unadjusted hazard ratio**	**95% CI**	**Adjusted hazard ratio**	**95% CI**
**Follow up TST outcome** *				
Negative < 10 mm	Reference		Reference	
Positive ≥ 10 mm	**61**.**28**	**7**.**95 - 472**.**38**	**58**.**08**	**7**.**23 - 435**.**21**
**Baseline TST outcome**				
Negative < 10 mm	Reference		Reference	
Positive ≥ 10 mm**	**1**.**30**	**0**.**37 - 4**.**63**	**4**.**70**	**0**.**56 - 39**.**41**
**Age group (years)**				
12-14	Reference		Reference	
15-16	1.58	0.39 - 6.33	1.58	0.39 - 6.33
17-18	3.49	1.02 - 11.94	2.49	0.67 - 9.28
**Sex**				
Male	Reference		Reference	
Female	0.27	0.08 – 0.95	0.32	0.09 - 1.17
**Education level**				
> 7 years	Reference		Reference	
None				
≤ 7 years	0.65	0.18 - 2.32	0.9	0.20 - 4.06
**TB contact during follow-up**				
No	Reference			
Yes	2.73	0.61 - 12.16		
**Cough during follow-up**				
<14 days	Reference			
None	0.71	0.14 - 3.54		
≥14 days	0.78	0.16 - 3.75		

Key: 95% CI = 95% Confidence Interval.

Note:

* One individual with TB during follow up had a positive TST of ≥10 mm at baseline;

**Those with a positive TST at baseline were not given TST again at 1 year and 2 years of follow up.

## Discussion

Among adolescents 12–18 years of age in a rural setting in Uganda, we found a prevalence of TB of 160/100,000 and incidence of 235/100,000 person years. The risk factors for prevalent TB were TB contact, cough of more than 14 days and being out of school whereas for incident TB; the only risk factor was a positive TST ≥ 10 mm.

All incident cases were detected at the scheduled 6 monthly visits and none through the health care system. This is probably due to active case finding leading to early detection of cases before they developed symptoms and presented to the health facilities [3]. All incident cases were culture positive but smear negative. This could have been due to sub-clinical colonization with mycobacteria. Although confidence intervals were wide, the TB prevalence of 160/100,000 in our sample was slightly lower than the estimated TB prevalence for all ages in Uganda (193/100,000). On the other hand the TB incidence of 235/100,000 person years was slightly higher than estimated incidence for all ages in Uganda (209/100,000) [1]. The observed differences in rates may be due to low HIV prevalence in this rural population, active TB case finding and adolescents having a lower TB incidence than the general population [1].

Furthermore, our study sample was mainly from a rural population, prevalence and incidence may have been much higher if an urban/peri-urban population was sampled. One of the few studies on TB prevalence in Uganda done in a slum area in Kampala city among chronic coughers ≥ 15 years reported a TB prevalence of 4,500/100,000 [12]. Future studies should target adolescents in crowded settlements in urban centers. Studies done in adult populations with high HIV prevalence in Africa have reported higher TB prevalence than our study [13-17].

We did not use CXR to screen TB suspects therefore we may have underestimated the TB prevalence and incidence in our population. In our study 4265 (85.3%) of the participants had no TB symptoms. Other prevalence surveys in the general population (usually among those aged over 15 years), that used CXR and/or symptoms to screen for TB have shown that 3852 (23%) of participants in Western Kenya [13] and 2972 (3.4%) in Vietnam [18] had CXR abnormalities suggestive of TB.

Risk factors for TB have been shown previously by others [9,13-15,17]. In our study, risk factors differed slightly between prevalent and incident cases. The high prevalence of TB among adolescents with TB contacts highlights the importance of contact tracing for increased TB case detection mainly in rural areas.

The strength of our study is the relatively large sample size and the fairly low loss to follow up rate (10.5%). This high cohort retention is good for potential future vaccine trials. A weakness of our study however was a relatively short follow up duration of 1.1 years. It is likely that a longer follow up would have yielded more TB cases. Furthermore we did not use chest X-ray as a screening tool. This may have resulted in underestimation of prevalent TB cases as previous studies documented an increase in number of TB cases when chest X-ray results were considered [19-24].

The baseline prevalence may have ‘cleared’ some incident cases, leading to an underestimation of the incidence. Moreover, we documented a high refusal rate from parents and adolescents. As we did not document possible reasons for refusal, we recommend future studies to explore this.

## Conclusion

The TB incidence among adolescents in this rural part of Uganda seemed too low for a phase III TB vaccine trial. A positive tuberculin test ≥10 mm, a history of TB contact and a cough ≥ 14 days are the major predictors for TB disease. However, the study demonstrated the capability of the study site to handle a large number of participants with minimal loss to follow-up which is important for future clinical trials. Improved contact tracing in TB program activities is likely to increase TB case detection among adolescents. Future studies should explore possible pockets of higher TB incidence in urban areas and among out of school youth.

## Competing interests

The authors declare that they have no competing interests.

## Authors’ contributions

JW: analyzed the data and wrote the paper, WS, MJ: managed the laboratory experiments, SV, AW, RC, WS, PM, and HMK: participated in critical review and manuscript writing, PM and HMK: conceived the idea. All authors participated in protocol development and read and approved the final version of the manuscript.

## Pre-publication history

The pre-publication history for this paper can be accessed here:

http://www.biomedcentral.com/1471-2334/13/349/prepub
